# The telomerase inhibitor Gno1p/PINX1 activates the helicase Prp43p during ribosome biogenesis

**DOI:** 10.1093/nar/gku357

**Published:** 2014-05-13

**Authors:** Yan-Ling Chen, Régine Capeyrou, Odile Humbert, Saïda Mouffok, Yasmine Al Kadri, Simon Lebaron, Anthony K. Henras, Yves Henry

**Affiliations:** Equipe labellisée Ligue Contre le Cancer, LBME, CNRS and Toulouse University, Toulouse 31062, France

## Abstract

We provide evidence that a central player in ribosome synthesis, the ribonucleic acid helicase Prp43p, can be activated by yeast Gno1p and its human ortholog, the telomerase inhibitor PINX1. Gno1p and PINX1 expressed in yeast interact with Prp43p and the integrity of their G-patch domain is required for this interaction. Moreover, PINX1 interacts with human PRP43 (DHX15) in HeLa cells. PINX1 directly binds to yeast Prp43p and stimulates its adenosine triphosphatase activity, while alterations of the G patch abolish formation of the PINX1/Prp43p complex and the stimulation of Prp43p. In yeast, lack of Gno1p leads to a decrease in the levels of pre-40S and intermediate pre-60S pre-ribosomal particles, defects that can be corrected by PINX1 expression. We show that Gno1p associates with 90S and early pre-60S pre-ribosomal particles and is released from intermediate pre-60S particles. G-patch alterations in Gno1p or PINX1 that inhibit their interactions with Prp43p completely abolish their function in yeast ribosome biogenesis. Altogether, our results suggest that activation of Prp43p by Gno1p/PINX1 within early pre-ribosomal particles is crucial for their subsequent maturation.

## INTRODUCTION

Ribosome biogenesis in eukaryotes involves the synthesis by ribonucleic acid (RNA) polymerase I of a polycistronic transcript precursor to the 18S, 5.8S and 25S/28S ribosomal RNAs (rRNAs) and by RNA polymerase III of a precursor to mature 5S rRNA. The RNA Pol I transcript contains external and internal spacer sequences that will need to be eliminated by sequential endo- and exoribonucleolytic cleavage steps to release the mature rRNAs [for reviews see (1–3)]. The nascent RNA Pol I transcript associates co-transcriptionally with a subset of ribosomal proteins, small nucleolar ribonucleoprotein particles (snoRNPs) and non-ribosomal proteins, so called because they are absent from mature cytoplasmic ribosomes. These assembly steps generate a nascent pre-ribosomal particle within which some specific nucleotides will be chemically modified mainly by box C/D snoRNPs that introduce methyl groups on the 2′ oxygen of specific ribose moieties and by box H/ACA snoRNPs that convert specific uridines into pseudouridines (4–6). In cases where the nascent pre-rRNA transcribed by RNA Pol I is not cleaved co-transcriptionally, a huge 90S pre-ribosomal particle will be generated. In *Saccharomyces cerevisiae*, its pre-rRNA component, called the 35S pre-rRNA, will undergo endonucleolytic cleavages at sites A0 and A1 in the 5′ external transcribed spacer followed by endonucleolytic cleavage at site A2 within the internal transcribed spacer 1. This A2 cleavage splits the evolving 90S particles into pre-40S and pre-60S pre-ribosomal particles, respectively precursor to the small and large ribosomal subunits. These particles will then follow independent maturation pathways while they move from the nucleolus to the nucleoplasm and then transit through the nuclear pores to enter the cytoplasm. Their maturation involves endo- and exoribonucleolytic processing and folding of their pre-rRNA component(s), assembly of ribosomal proteins and ill-defined structural rearrangements coupled with dynamic association and dissociation of scores of non-ribosomal proteins (7).

Close to 200 non-ribosomal proteins that transiently associate with various pre-ribosomal particles have been identified in *S. cerevisiae*. Some of these belong to well-defined enzyme families, such as ATPases, GTPases, kinases, nucleotide modifying enzymes, endo- and exoribonucleases and RNA helicases, while others do not bear any obvious sequence signature (8). The most abundant group is that of RNA helicases. No less than 19 non-ribosomal proteins belonging to the SFII family of RNA helicases are involved in ribosome biogenesis in yeast (9,10). Most of these are essential for viability and ribosome synthesis, indicating that they do not play redundant functions. RNA helicases are believed to couple nucleoside triphosphate [usually adenosine triphosphate (ATP)] hydrolysis to structural rearrangements of RNA/RNA and/or RNA/protein complexes (11). RNA helicases have been assigned several roles during ribosome biogenesis, such as facilitating the folding of (pre-)rRNA sequences, unfolding secondary structures in spacer sequences to facilitate their degradation by exoribonucleases, promoting the dissociation of box C/D snoRNAs or non-ribosomal proteins from pre-rRNAs (9,10). Several reports support these proposals. For example, the Mtr4p/Dob1p RNA helicase is required for 7S pre-rRNA processing and could participate in the unfolding of internal transcribed spacer 2 sequences to facilitate their exonucleolytic digestion by the exosome (12,13). Depletion of the RNA helicases Dbp4p and Rok1p or alterations of key catalytic residues of these enzymes cause the specific retention within early pre-ribosomal particles of the U14 and snR30 snoRNAs, respectively (14,15). These data suggest that Dbp4p and Rok1p participate in the unwinding of the U14 and snR30 snoRNAs from the pre-rRNA, although direct demonstration that this is the case is still lacking.

One of the most intriguing RNA helicases involved in ribosome biogenesis is the DEAH box protein Prp43p. Yeast Prp43p was first characterized as a splicing factor, required for the release of the U2, U5 and U6 spliceosomal snRNPs from the spliced out intron lariat, thus allowing snRNP recycling and lariat linearization (16–18). It was later shown that Prp43p is also an ubiquitous component of yeast pre-ribosomal particles (19–21). Prp43p associates with 90S, pre-40S and pre-60S pre-ribosomal particles. Depletion of Prp43p leads to a transient accumulation of the 35S pre-rRNA and reduced accumulation of all downstream pre-rRNA intermediates, resulting in impaired production of both the small and large ribosomal subunits. It is likely that Prp43p intervenes at distinct steps of the ribosome biogenesis pathway (22) and could be targeted to and/or activated within different pre-ribosomal particles by different co-factors. Three such potential co-factors termed Ntr1p, Pfa1p and Gno1p were identified using double-hybrid screens, immunoprecipitations or tandem affinity purifications (TAPs) (18,20). Pfa1p is by far the most abundant polypeptide among the proteins retained on the calmodulin column after TAP purification of Prp43p (20), suggesting that at steady state, the Prp43p/Pfa1p complex predominates. Moreover, Gno1p, which is also co-purified with Prp43p-TAP, exhibits a positive double-hybrid interaction with Prp43p (20). Ntr1p, Pfa1p and Gno1p all contain a so-called G-patch domain (23), characterized by the presence of conserved glycines, hence the name. The role of Ntr1p with respect to Prp43p was characterized first. Ntr1p binds directly to Prp43p, stimulates its helicase activity *in vitro* and allows Prp43p to disassemble the U2, U5 and U6 spliceosomal snRNPs from the spliced out intron lariat in splicing extracts (18,24,25). Pfa1p was also shown to bind directly to Prp43p *in vitro* and to stimulate its ATPase and helicase activities (26). Pfa1p interacts with several pre-ribosomal particles but at steady state, it is found mostly associated with 20S pre-rRNA, suggesting that it is involved in pre-40S pre-ribosomal particle maturation (20). Lack of Pfa1p causes only subtle phenotypes, but the combined absence of Pfa1p and Ltv1p, another component of pre-40S particles, leads to a severe growth defect and accumulation of 20S pre-rRNA in the cytoplasm (27). The phenotypes of cells lacking Pfa1p and Ltv1p can be partially corrected by overexpression of the Nob1p endonuclease (27). This enzyme converts 20S pre-rRNA into 18S rRNA by cleaving the D site, generating the mature 3′ end of 18S rRNA (27,28). Hence it is proposed that activation of Prp43p by Pfa1p is required for efficient 20S pre-rRNA processing, probably because it allows a conformational rearrangement of pre-40S particles which, directly or indirectly, facilitates D site cleavage by Nob1p.

Gno1p, the third G-patch partner of yeast Prp43p, is also involved in ribosome biogenesis in yeast (29). No role in pre-messenger RNA (mRNA) splicing has yet been described for Gno1p. Whether Gno1p is a direct co-activator of Prp43p has remained an unsolved issue, mainly because we have so far failed to obtain purified recombinant Gno1p necessary for unambiguous protein–protein interaction studies. To circumvent this problem, we have turned to PINX1, the human ortholog of yeast Gno1p, that was previously characterized as a telomerase inhibitor (30). We show that PINX1, which can functionally replace Gno1p in yeast (29), interacts with Prp43p in yeast and directly binds to Prp43p *in vitro* to stimulate the ATPase activity of the helicase. Our data further demonstrate that Gno1p is required for the normal accumulation of pre-40S and intermediate pre-60S pre-ribosomal particles and suggest that Prp43p activation by Gno1p/PINX1 is required for the production and/or stability of pre-40S and intermediate pre-60S pre-ribosomal particles.

## MATERIALS AND METHODS

### Yeast strains and plasmids

The MW3627 *S. cerevisiae* *Δgno1* strain (*MATα ura3–52 his3-Δ200 trp1-Δ63 LEU2 gno1-Δ16–769::kanMX4*) and its congenic wild-type strain MW3628 (*MATα ura3–52 his3-Δ200 trp1-Δ63 leu2-Δ1 GNO1*) were obtained from Michel Werner.

A strain expressing HA-tagged Gno1p from the *GAL1* promoter (*GAL1–3HA-GNO1*) was constructed by transforming strain BY4741 (*MATa his3Δ1 leu2Δ0 met15Δ0 ura3Δ0*) (Open Biosystems) with a polymerase chain reaction (PCR) fragment produced using plasmid pFA6a-kanMX6-PGAL1–3HA (31) and oligonucleotides OHA403 (5′AAAAAAAAAAAATATGAAAAATTTTGTTCTGAAAGAAGCGATGAGATGAGGAATTCGAGCTCGTTTAAAC3′) and OHA404 (5′CTGGGGTCTAAACCAAACCGCTGTTTGGTTCTTGTAGCTGCCAAACCCATGCACTGAGCA GCGTAATCTG 3′).

Strains expressing TAP-tagged versions of Utp15p, Utp22p, Pwp2p, Utp6p, Utp13p, Utp18p, Utp21p, Prp43p, Pfa1p, Rio2p, Ssf1p, Nog2p, Rix1p and Arx1p were constructed by transformation of strain MW3628 with PCR fragments obtained using genomic deoxyribonucleic acids (DNAs) extracted from strains expressing the corresponding TAP-tagged protein [Open Biosystems collection, with the exception of the Prp43p-TAP (20) and Ssf1p-TAP (32) expressing strains] and the following oligonucleotides:

UTP15TAPF (5′GCGAAAAGTAATAACTTCCAGAG3′) and UTP15TAPR (5′GAGTATTCGAAAATGTTCAAATAC3′),
UTP22TAPF (5′GCTAAAGTAGCCGTGAATCTTTTAC3′) and UTP22TAPR (5′CGGTAACAGTTTAATGAAATC3′),
PWP2TAPF (5′CAATAAAGTCTATGAAGCCATACC3′) and PWP2TAPR (5′CATTAAACGTCCTTTGAAATATACGG3′),
UTP6TAPF (5′GACATTGCATTAACTATTTTCGATGTG3′) and UTP6TAPR (5′GGTCGAATTCTGCACATAGGTCGAAC3′),
UTP13TAPF (5′GAAATAGAAGAAGAACAAGAAAAAGC3′) and UTP13TAPR (5′GAGAAAACATGATCACTTTTACCATTTTC3′),
UTP18TAPF (5′GACTTGAATAAGAATGGCCATGTTATAAG3′) and UTP18TAPR (5′CGAAAAACATAGGTGAACCTAAAACC3′),
UTP21TAPF (5′GGGGATGAAGCCTCTGTCAGAGAAGGC3′) and UTP21TAPR (5′CGAGTTGCGATTCTTTTCTATCAGTTGG3′),
PRP43TAPF (5′GTGGTGCCGTGACCACTATCTAAATTAC3′) and PRP43TAPR (5′GTCACTTAGATAAAACATGATTGCTAC3′),
PFA1TAPF (5′GATGAATTTGAGTCCTTTTTGTCTAGG3′) and PFA1TAPR (5′GATGACATCTTCAGGGTTAACGAATAAAGG3′),
RIO2TAPF (5′GATTTTCCACAATGTATATCTATTC3′) and RIO2TAPR (5′GCTACCCGTCAAATGCAAGACGAG3′),
SSF1TAPF (5′CCCAAAGAACACCGGTCGAAAAGAAGGATAATAAAG3′) and SSF1TAPR (5′CAGGGTTTCCTAGCAAACTTGCAGGATTTTGATAG3′),
NOG2TAPF (5′CCAAACACTGGTAAATCGTCCATCATTAACAC3′) and NOG2TAPR (5′GCTAATACCCTCGGTTGTACATTCATAG3′),
RIX1TAPF (5′GATATGTTTGAATTACTGTGTCATCC3′) and RIX1TAPR (5′GAGAATGCTAGCCAAGATAACTCCCG3′) and
ARX1TAPF (5′CATTTAGACTTGGGATGAGTGAGATTTCC3′) and ARX1TAPR (5′CTATCTGGAGTTCAAGGTTCCGTCAAATC3′).

A strain expressing a ZZ-tagged version of Npa1p was constructed by transformation of strain MW3628 with a PCR fragment obtained using genomic DNA extracted from a Npa1p-ZZ expressing strain (32) and oligonucleotides NPA1ZZF (5′GAAATTGATATGGAAATATACTATGG3′) and NPA1ZZR (5′GGAAGGCGCGAAACCAACGCTGACTG3′).

Strains expressing Gno1p-HA and the above-listed tagged ribosome biogenesis factors were obtained by transforming the strains expressing the tagged ribosome biogenesis factors just described with a *GNO1-HA-TRP1* cassette PCR amplified using plasmid pFA6a-3HA-TRP1 (31) and oligonucleotides R1GNO1HA (5′GGGGAAGAATTTTTCCCCACTCTATATATCTTGCTGCGTGTGCAGACTGGCCAGCTGCTCGAATTCGAGCTCGTTTAAAC5′) and F2GNO1HA (5′GAGGGCCGCGTTGATGGACTCCAAGGCACTGAATGAGATCTTTATGATAACAAACGACCGGATCCCCGGGTTAATTAA3′).

A *Δgno1*/*PRP43-TAP* strain was produced by transforming the *Δgno1* strain MW3627 with the *PRP43-TAP* PCR cassette obtained as described above.

*Δgno1* and *Δgno1*/*PRP43-TAP* strains expressing proteins Gno1p-HA, Gno1pGm1-HA, Gno1pGm2-HA, PINX1-HA, PINX1Gm1-HA and PINX1Gm2-HA were obtained by transformation of the *Δgno1* and *Δgno1/PRP43-TAP* strains with plasmids pFH170, pFH174, pFH175, pFH172, pFH178 and pFH179, respectively. Plasmid pFH170 was produced by inserting into SphI-digested pHA113 (33) a SphI-digested *GNO1-HA* PCR cassette obtained by PCR amplification performed with plasmid pFH152 and oligonucleotides 5′SphI-GNO1 (5′GGGGGGCATGCATGGGTTTGGCAGCTACAAGAACCAAACAGC3′) and 3′GNO1–2HA-SphI (5′GGGGGGCATGCTTATCATGCATAGTCCGGGACGTCATACGGATAGCCCGCATAGTCAGGAACATCGTATGGGTAGTCGTTTGTTATCATAAAGATCTCAT3′). Plasmid pFH152 was obtained by inserting into SphI cut pHA113 (33) a SphI-digested PCR cassette of the *GNO1* ORF lacking a stop codon amplified using oligonucleotides Ggpatchinpha01 (5′GGGGGGCATGCATGGGTTTGGCAGCTACAAGAACC3′) and Gno1in3202 (5′CCCCCGCATGCGTCGTTTGTTATCATAAAGATCTC3′). Plasmids pFH174 and pFH175 were obtained by replacing a *GNO1* ORF BamHI fragment from plasmid pFH170 by the equivalent mutated BamHI fragments extracted from plasmids pYLC1 and pYLC2, respectively. Plasmids pYLC1 and pYLC2 were obtained by replacing the *GNO1* ORF Eco130I/XhoI fragment of pFH152 by the equivalent mutated fragments extracted from plasmids pSL59 and pSL63, respectively, encoding altered versions of Gno1p featuring the L33A-L44A-L67A (pSL59) or L33A-G37E-G41E-G43E-L44A-G45E-L67A (pSL63) amino acid substitutions with the Gno1p G patch. Plasmid pSL59 was obtained by replacing the *GNO1* ORF XhoI/BglII fragment from pSL21 by the equivalent mutated fragment from pSL43 encoding Gno1p L33A-L44A-L67A. Plasmid pSL21 was obtained by inserting a SmaI *GNO1* ORF cassette into pGEX-2TK plasmid digested by SmaI. Plasmid pSL63 was produced by mutagenic reverse PCR amplification of plasmid pSL59 using mutagenic oligonucleotides Gno1mut3G3L-01 (5′TCGGGCACCAATTTGCAGAAAAGTTTGAATGGAAACCCGAAATGGAAGCGGAGTTATCCCCCATGAATTCGAACACTTCG3′) and Gno1mut3G3L-02 (5′CGAAGTGTTCGAATTCATGGGGGATAACTCCGCTTCCATTTCGGGTTTCCATTCAAACTTTTCTGCAAATTGGTGCCCGA3′). Plasmid pFH172 was obtained by inserting into SphI-digested pHA113 a SphI-digested *PINX1-HA* PCR cassette obtained by PCR amplification performed with plasmid pRC42 and oligonucleotides 5′PINX1-SphI (5′GGGGGGCATGCATGTCTATGCTGGCTGAACGTCGGCGGAAGCAGAAGTGGGCTGTGGATCCTC3′) and 3′PINX1–2HA-SphI (5′GGGGGGCATGCTTATCATGCATAGTCCGGGACGTCATACGGATAGCCCGCATAGTCAGGAACATCGTATGGGTATTTGGAATCTTTCTTCTTCTTCTTTT3′). Plasmid pRC42 was obtained by inserting into XbaI/XhoI-digested pSCodon1 a XbaI/XhoI-digested *PINX1-HIS* PCR cassette amplified using oligonucleotides RC92–5′ (5′GGGGGTCTAGATTTAAGAAGGAGATATACATATGTCTATGCTGGCTGAACGTCGGCGG3′) and RC93–3′ (5′GGGGGCTCGAGTTAATGGTGATGGTGATGGTGTTTGGAATCTTTCTTCTTCTT3′). Plasmids pFH178 and pFH179 directing expression in yeast of HA-tagged PINX1-L34A-L45A or PINX1-L34A-G38E-G42E-G44E-L45A-G46E were produced by reverse PCR amplification of plasmid pFH172 using mutagenic oligonucleotides PINX1mut1 (5′TCCAAGTTTGGCCAGCGGATGGCAGAGAAGATGGGGTGGTCTAAAGGAAAGGGTGCAGGGGCTCAGGAGCAAGGAGCCAC3′) and PINX1mut2 (5′GTGGCTCCTTGCTCCTGAGCCCCTGCACCCTTTCCTTTAGACCACCCCATCTTCTCTGCCATCCGCTGGCCAAACTTGGA3′), or PINX1mut3 (5′TCCAAGTTTGGCCAGCGGATGGCAGAGAAGATGGAGTGGTCTAAAGAAAAGGAAGCAGAGGCTCAGGAGCAAGGAGCCAC3′) and PINX1mut4 (5′GTGGCTCCTTGCTCCTGAGCCTCTGCTTCCTTTTCTTTAGACCACTCCATCTTCTCTGCCATCCGCTGGCCAAACTTGGA3′), respectively.

### *Escherichia coli* strains and plasmids

*E. coli* strains directing expression of Prp43p-HIS, GST-PINX1-HIS and GST-PINX1Gm-HIS were obtained by transforming *E. coli* BL21 with plasmids pSL18 (26), pRC43 and pFH177, respectively. pRC43 was obtained by inserting into EcoRI/NotI-digested pGEX-4T1 an EcoRI/NotI-digested *PINX1-HIS* PCR cassette obtained by amplifying the *PINX1* ORF using oligonucleotides RC94 (5′GGGGGGAATTCATGTCTATGCTGGCTGAACGTCGGCGG3′) and RC95 (5′GGGGGGCGGCCGCTTAATGGTGATGGTGATGGTGTTTGGAATCTTTCTTCTTCTT3′). pFH177 was obtained by reverse PCR amplification of plasmid pRC43 using mutagenic oligonucleotides PINX1mut3 and PINX1mut4.

### Recombinant protein purification

Transformed *E. coli* BL21 λDE3 bacteria were grown at 37°C in Luria-Bertani (LB) medium supplemented with 100 μg/ml ampicillin to an A_600_ of 0.6. Recombinant protein expression was then induced with 1-mM isopropyl β-D-thiogalactoside, and culturing was continued overnight at 18°C. Bacteria were harvested by centrifugation (6000 g, 20 min, 4°C), resuspended in 50-mM Tris-HCl (pH 7.5), 500-mM sodium chloride, 0.2% Triton-X100, 10% glycerol, 10-mM imidazole, 5-mM 2-mercaptoethanol and frozen at −80°C. Bacteria were thawed and sonicated on ice in the presence of a complete ethylenediaminetetraacetic acid (EDTA)-free protease inhibitor cocktail (Roche). Following centrifugation (40 000 g, 30 min, 4°C), the cleared lysate was subjected to affinity chromatography using a HisTrap HP column (GE Healthcare) and proteins were eluted with a linear gradient of imidazole. Fractions containing histidine (His)-tagged proteins were identified by sodium dodecyl sulphate-polyacrylamide gel electrophoresis (SDS-PAGE) and gel staining with Coomassie brilliant blue. Positive fractions were pooled and the proteins of interest were purified by size-exclusion chromatography with a Superdex 200 gel filtration column (GE Healthcare) in 50-mM Tris-HCl (pH 7.5), 500-mM sodium chloride, 0.2% Triton X100, 10% glycerol and 5-mM 2-mercaptoethanol. Fractions were subjected to SDS-PAGE and proteins were detected by staining with Coomassie brilliant blue. The fractions containing the monomeric form of the proteins were stored at –80°C.

### Pull-down assays

A total of 2 μg of each recombinant purified protein were mixed in the indicated combinations and incubated with 50 μl of stacked protein A/protein G Sepharose beads (GE Healthcare) in 500-μl IP buffer [25-mM Tris-HCl (pH 8.0), 300-mM KCl, 5-mM MgCl_2_, 10% glycerol, 0.1% NP-40 and 0.5-mM dithiothreitol (DTT)] supplemented with complete EDTA-free protease inhibitor cocktail (Roche) with gentle shaking for 1 h at 4°C. The supernatant was collected and incubated with 50 μl of stacked protein A/protein G Sepharose beads coated with anti-Prp43p antibodies (20). Incubation of protein A/protein G Sepharose beads and pre-cleared His-tagged recombinant proteins was performed for 2 h at 4°C with gentle shaking. The supernatant was collected and proteins were precipitated with TCA. Beads were washed four times with 1-ml IP buffer and proteins retained on the beads were eluted with 50-μl SDS-PAGE loading buffer [100-mM Tris-HCl (pH 6.8), 4% SDS, 20% glycerol, 200-mM DTT and 0.04% bromophenol blue]. Proteins were analyzed by western blotting using His mAb HRP conjugate (Clontech).

### Immunoprecipitation experiments using HeLa cell extracts

5–10.10^6^ HeLa cells were rinsed with ice-cold phosphate buffered saline, scraped and recovered by centrifugation. Cell pellets were resuspended with 500-μl IP buffer [50-mM Tris-HCl (pH 8.0), 5-mM MgAc, 200-mM NaCl, 0.5% NP-40] supplemented with complete EDTA-free protease inhibitor cocktail (Roche) and were disrupted by sonication (pulses of 30 s at level medium separated by 30-s incubations on ice during 5 min), using a Bioruptor (Diagenode). Cell extracts were clarified by centrifugation at 16 000 g and 4°C for 10 min and incubated with 15 μl of protein A/protein G Sepharose beads (GE Healthcare) coated with anti-DHX15 or anti-PINX1 antibodies for 2 h at 4°C with gentle shaking. Sepharose beads were rinsed six times with 1 ml of IP buffer and were resuspended with 25 μl of 2X SDS gel-loading buffer [100-mM Tris–HCl (pH 6.8), 4% SDS, 20% glycerol, 200-mM DTT, 0.2% bromophenol blue]. Immunoprecipitated proteins were analyzed by western blot using rabbit anti-DHX15 antibodies (ab70454; Abcam) or goat anti-PINX1 antibodies (ab99112; Abcam) used at 1000-fold dilution.

### Pulse-chase analysis

Approximately 8.10^7^ cells of *S. cerevisiae Δgno1* and congenic wild-type strain growing exponentially in adenine-free minimal medium at 30°C were labeled for 2 min with [8–^3^H] adenine (1 mCi/ml). One milliliter cold adenine (1 mg/ml) was then added, cells were harvested after 1, 2, 5, 15, 30, 60, 90 and 120 min and cell pellets were immediately frozen in liquid nitrogen. Purified RNAs were separated on a 1.2% agarose gel and transferred to Amersham Hybond N^+^ membrane (GE Healthcare). Labeled RNAs were detected by autoradiography using BioMax KODAK™ MR films inserted in a KODAK™ BioMax® TranScreen LE.

### ATPase assays

The ATPase activity of the proteins (0.1 μM) was measured in a 5-μl reaction volume containing 25-mM Tris-acetate (pH 8.0), 10-mM Mg(CH_3_COO)_2_, 0.2-mM DTT, 100-μg/ml bovine serum albumin (Sigma), 0.6 μCi/μl [α-^32^P] ATP and unlabeled ATP at 100 μM. The reaction mixtures were incubated at 30°C for 0, 5, 10, 30 and 60 min, stopped on ice and 1 μl from each sample was analyzed by thin-layer chromatography on polyethyleneimine-cellulose plates (Merck) using 0.75-M KH_2_PO_4_ as migration buffer. The plates were dried and radioacitvity was quantified on a Fuji BAS 3000 Phosphorimager. When needed, reactions were performed in the presence of ribonuclease (RNase) (1 mg/ml) or 150 μM of total yeast RNA. For the latter condition, unlabeled ATP is added at a final concentration of 200 μM.

### Sedimentation on sucrose gradients, Immunoprecipitations from yeast extracts, Northern and primer extensions

Sedimentation on sucrose gradients was performed as described in (34), except that 10–50% sucrose gradients were used. Immunoprecipitations of TAP-tagged proteins from yeast extracts, northern analyses and primer extensions were performed as described in (34). Immunoprecipitations of HA-tagged proteins from yeast extracts were performed as for TAP-tagged proteins except that EZview Red Anti-HA Affinity Gel (Sigma, E6779) was used instead of IgG Sepharose 6 fast flow.

## RESULTS

### Conserved interactions between Gno1p or PINX1 and the helicase Prp43p/DHX15 in yeast and human cells

**Figure 1.**
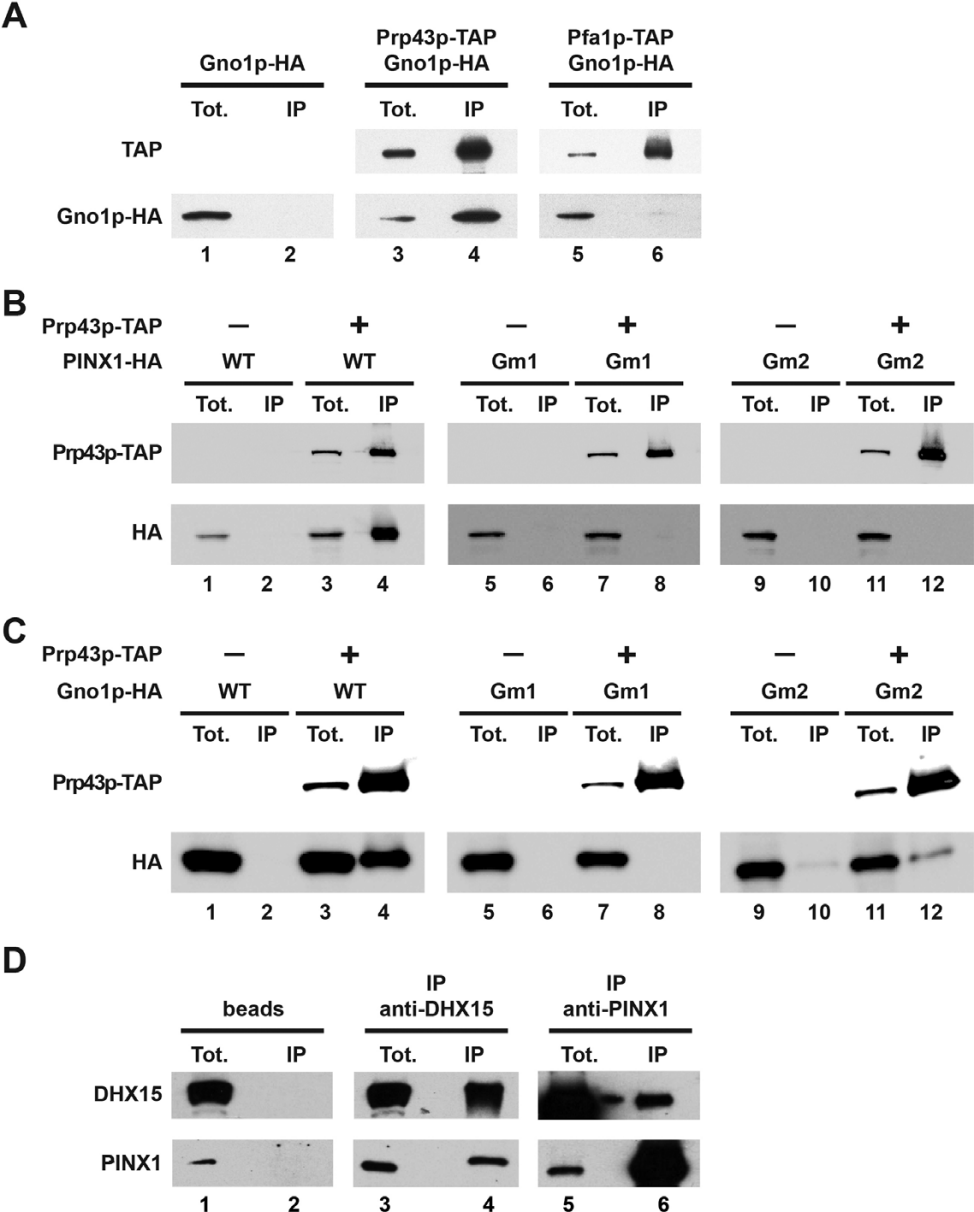
Interactions between Prp43p or DHX15 and the G-patch proteins Gno1p or PINX1. (**A**) Gno1p-HA associates with Prp43p-TAP. Immunoprecipitations have been performed using IgG sepharose beads and cells expressing Gno1p-HA (lanes 1 and 2), Prp43p-TAP and Gno1p-HA (lanes 3 and 4) or Pfa1p-TAP and Gno1p-HA (lanes 5 and 6). Proteins have been extracted from the pellets (lanes IP) or from 1/320th of the input extracts (lanes Tot.), separated by SDS-PAGE and transferred to nitrocellulose. TAP-tagged proteins have been detected using rabbit PAP and HA-tagged Gno1p using anti-HA antibodies. (**B**) Human PINX1-HA interacts with Prp43p-TAP in yeast and the integrity of PINX1 G patch is important for this interaction. Immunoprecipitations have been performed as described in (A) using yeast cells expressing PINX1-HA (lanes 1 and 2), Prp43p-TAP and PINX1-HA (lanes 3 and 4), PINX1Gm1-HA (lanes 5 and 6), Prp43p-TAP and PINX1Gm1-HA (lanes 7 and 8), PINX1Gm2-HA (lanes 9 and 10) and Prp43p-TAP and PINX1Gm2-HA (lanes 11 and 12). PINX1Gm1-HA features the L34 to A and L45 to A substitutions, while PINX1Gm2-HA features the L34 to A, G38 to E, G42 to E, G44 to E, L45 to A and G46 to E substitutions. (**C**) Gno1p-HA interacts with Prp43p-TAP in yeast and the integrity of Gno1p G patch is important for this interaction. Immunoprecipitations have been performed as described in (A) using yeast cells expressing Gno1p-HA (lanes 1 and 2), Prp43p-TAP and Gno1p-HA (lanes 3 and 4), Gno1pGm1-HA (lanes 5 and 6), Prp43p-TAP and Gno1pGm1-HA (lanes 7 and 8), Gno1pGm2-HA (lanes 9 and 10) and Prp43p-TAP and Gno1pGm2-HA (lanes 11 and 12). Gno1pGm1-HA features the L33 to A, L44 to A and L67 to A substitutions, while Gno1pGm2-HA features the L33 to A, G37 to E, G41 to E, G43 to E, L44 to A, G45 to E and L67 to A substitutions. (**D**) PINX1 interacts with DHX15 in HeLa cells. Immunoprecipitations have been performed with HeLa cell extracts using an anti-DHX15 serum (lanes 3 and 4), an anti-PINX1 serum (lanes 5 and 6) or no serum (lanes 1 and 2). Proteins extracted from the pellets (lanes IP) or from 1/30th (top lane) or 1/250th (bottom lane) of the input extracts (lanes Tot.) have been processed as described in (A) and detected using the anti-DHX15 or anti-PINX1 sera.

We have previously shown that the yeast G-patch protein Gno1p is physically associated with Prp43p. Indeed Gno1p exhibits a positive double-hybrid interaction with yeast Prp43p and Gno1p is found associated with Prp43p after its TAP (20). We have now further confirmed these results by showing that HA-tagged Gno1p expressed in yeast is co-precipitated with Prp43p-TAP using IgG-sepharose beads (Figure 1A, lanes 3 and 4). In contrast, Gno1p-HA is not co-precipitated with Pfa1p-TAP, the other G-patch partner of Prp43p implicated in ribosome biogenesis (Figure 1A, lanes 5 and 6). This latter result suggests that the interactions between Prp43p and Gno1p on the one hand and between Prp43p and Pfa1p on the other hand are mutually exclusive. It has previously been shown that the putative human ortholog of yeast Gno1p, the telomerase inhibitor PINX1, can restore the growth and normal rRNA accumulation of cells lacking Gno1p (29). We have now assessed whether PINX1 expressed in yeast can also interact with yeast Prp43p. Indeed this is the case, since we find that HA-tagged PINX1 expressed in *Δgno1* cells is efficiently co-precipitated with Prp43p-TAP from total yeast extracts (Figure 1B, lanes 3 and 4).

With the view of determining whether the G-patch domain is important for the interaction of Gno1p or PINX1 with Prp43p in yeast, we generated yeast strains expressing Prp43p-TAP and altered versions of Gno1p or PINX1, termed Gno1pGm1-HA, Gno1pGm2-HA, PINX1Gm1-HA and PINX1Gm2-HA, featuring substitutions of conserved amino acids within the G-patch domain. Gno1pGm1-HA features the L33 to A, L44 to A and L67 to A substitutions, while Gno1pGm2-HA features the L33 to A, G37 to E, G41 to E, G43 to E, L44 to A, G45 to E and L67 to A substitutions. PINX1Gm1-HA features the L34 to A and L45 to A substitutions, while PINX1Gm2-HA features the L34 to A, G38 to E, G42 to E, G44 to E, L45 to A and G46 to E substitutions. The co-precipitation with Prp43p-TAP of these altered proteins is inhibited (Figure 1B and C, lanes 5 to 12), showing that the integrity of the G patch is essential for the interaction of Gno1p or PINX1 with Prp43p in yeast.

Finally, we have tested whether PINX1 interacts with human PRP43, also called DHX15, in HeLa cells. Our data demonstrate that PINX1 is efficiently co-precipitated with DHX15 from HeLa cell extracts using a polyclonal anti-DHX15 serum (Figure 1D, lanes 3 and 4), while conversely, DHX15 is efficiently co-precipitated with PINX1 when an anti-PINX1 polyclonal serum is used (Figure 1D, lanes 5 and 6). Altogether our data show that the Prp43p/Gno1p interactions are conserved in yeast and human.

### Human PINX1 binds directly to yeast Prp43p and stimulates Prp43p ATPase activity *in vitro*

**Figure 2.**
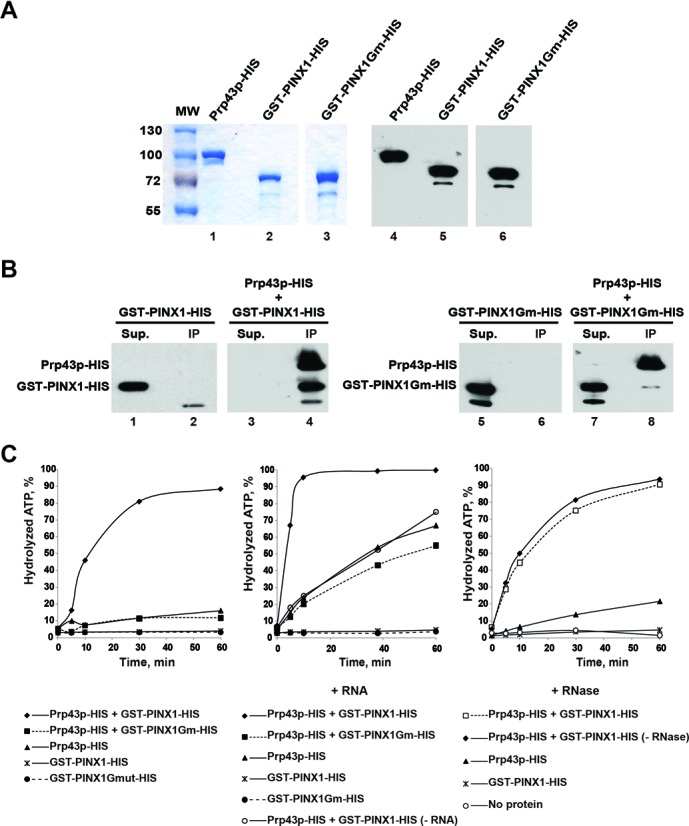
Human PINX1 binds directly to yeast Prp43p and stimulates Prp43p ATPase activity. (**A**) Purification and analysis of recombinant proteins. Prp43p-HIS (lanes 1 and 4), GST-PINX1-HIS (lanes 2 and 5) and GST-PINX1Gm-HIS (lanes 3 and 6) were expressed in *E. coli* and purified in two steps (affinity purification on a nickel column followed by size-exclusion chromatography). Purified proteins were submitted to SDS-PAGE and analyzed by Coomassie blue staining (left panel) or western using anti-histidine antibodies (right panel). (**B**) Pull-down assays. Purified recombinant GST-PINX1-HIS and GST-PINX1Gm-HIS were incubated with Prp43p-HIS (lanes 3–4 and 7–8, respectively). In control experiments, Prp43p-HIS was omitted (lanes 1, 2, 5 and 6). Immunoprecipitations were then carried out using anti-Prp43p antibodies. Proteins extracted from the pellets (lanes IP) or TCA precipitated from the supernatants (lanes Sup.) were subjected to SDS-PAGE, transferred to nitrocellulose membranes and detected by western using anti-histidine antibodies. (**C**) ATPase assays. [α-^32^P]-ATP was incubated with the indicated proteins and when mentioned with total yeast RNA (150 μM) or RNase (1 mg/ml). Note that assays were performed in the presence of 100-μM cold ATP, except when RNA was added, in which case the cold ATP concentration was increased to 200 μM. The percentage of hydrolyzed ATP is plotted with respect to time (in minutes).

To assess whether Prp43p and Gno1p directly interact, we decided to carry out *in vitro* pull-down assays using recombinant purified proteins. Unfortunately, we failed to obtain sufficient expression of histidine (HIS) or glutathione S-transferase (GST) tagged Gno1p in *E. coli* for purification purposes, while maltose binding protein (MBP) tagged Gno1p was purified in the form of large aggregates. However, we could express in *E. coli* and purify soluble HIS- and GST-tagged human PINX1 (Figure 2A, lanes 2 and 5). Since human PINX1 is able to complement the growth and rRNA accumulation defects of yeast *Δgno1* cells and interacts with yeast Prp43p *in vivo*, we felt that cross-species binding studies involving yeast Prp43p and human PINX1 were entirely justified. Purified recombinant yeast Prp43p-HIS and human GST-PINX1-HIS were mixed and Prp43p-HIS was precipitated using an anti-Prp43p polyclonal serum. Western analysis using anti-HIS tag antibodies shows that GST-PINX1-HIS is co-precipitated together with Prp43p-HIS (Figure 2B, lanes 3 and 4), demonstrating that the two proteins can directly interact *in vitro*. The GST tag is not responsible for this interaction since the unrelated GST-tagged OvoAC protein was not co-precipitated with Prp43p-HIS (Supplementary Figure S1). To assess whether the G-patch domain of PINX1 is involved in the interaction, we performed the same binding and pull-down experiments using an altered PINX1 protein, termed GST-PINX1Gm-HIS, featuring the L34 to A, G38 to E, G42 to E, G44 to E, L45 to A, and G46 to E amino acid substitutions within the G patch (Figure 2A, lanes 3 and 6). This altered version of PINX1 failed to interact with Prp43p-HIS (Figure 2B, lanes 7 and 8). We conclude that the integrity of the G-patch domain of PINX1 is required for its binding to Prp43p.

We have previously shown that the G-patch protein Pfa1p binds directly to Prp43p *in vitro* and stimulates its ATPase activity (26). We tested whether likewise, GST-PINX1-HIS is able to stimulate Prp43p-HIS ATPase activity. On its own, Prp43p is a rather feeble ATPase [(26) and Figure 2C, left panel]. Addition of purified GST-PINX1-HIS to Prp43p-HIS greatly stimulates the ATPase activity (Figure 2C, left panel). After 60 min of incubation under our assay conditions, Prp43p-HIS hydrolyzes 9-fold more ATP when GST-PINX1-HIS is added. Note that on its own, GST-PINX1-HIS displays no ATPase activity (Figure 2C, left panel). Furthermore, this stimulation is not due to contaminating RNAs present in the GST-PINX1-HIS preparation, as RNase treatment did not diminish its stimulatory potential (Figure 2C, right panel). As reported previously (26), the ATPase activity of Prp43p-HIS can also be stimulated by addition of RNA (Figure 2C, central panel). Addition of RNA and GST-PINX1-HIS leads to a greater stimulation of the ATPase activity than the same amounts of each compound added separately, demonstrating that RNA and GST-PINX1-HIS can cooperate in activating Prp43p and suggesting that the binding sites on Prp43p for RNA and the G-patch protein are distinct. In contrast to GST-PINX1-HIS, GST-PINX1Gm-HIS failed to activate Prp43p-HIS, whether or not RNA was present (Figure 2C). Altogether, our data show that GST-PINX1-HIS binds directly to yeast Prp43p resulting in a stimulation of the ATPase activity of Prp43p. The integrity of the G patch of GST-PINX1-HIS is required for its binding to Prp43p, strongly suggesting that the two proteins interact via the G patch.

### Yeast Gno1p is required for normal accumulation of pre-40S and intermediate pre-60S particles

**Figure 3.**
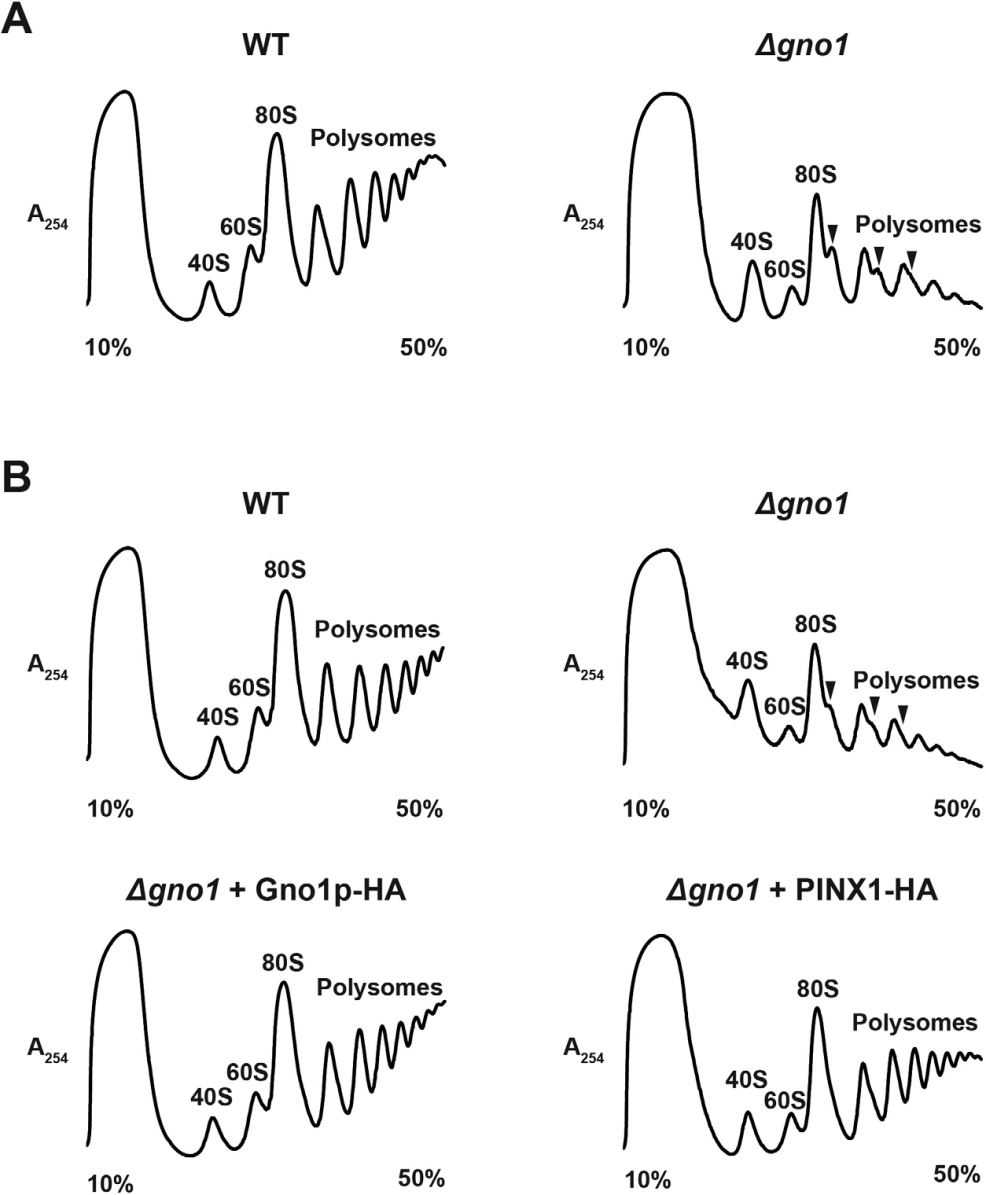
Gno1p is required for the normal accumulation of the free large ribosomal subunit. (**A**) Accumulation of free ribosomal subunits and polysomes in *Δgno1*compared to WT cells. Extracts from wild type (WT) (left panel) and *Δgno1* (right panel) cells grown in yeast extract peptone (YP) glucose medium were centrifuged on 10–50% sucrose gradients at 39 000 revolutions per minute in a SW41Ti rotor. The abundance of free 40S, 60S ribosomal subunits, 80S ribosomes and polysomes was assessed by recording the ultraviolet absorbance at 254 nm in the successive gradient fractions. (**B**) Accumulation of free ribosomal subunits and polysomes in *Δgno1* cells expressing Gno1p-HA (bottom left panel) or PINX1-HA (bottom right panel). As in (A), except that cells were grown in supplemented yeast nitrogen medium (YNB Glu). Profiles obtained with WT (upper left panel) and *Δgno1* cells (upper right panel) are shown as controls.

Guglielmi and Werner have previously shown that lack of Gno1p leads to a deficit in both 18S and 25S rRNAs (29). To assess the relative impairment in the accumulation of the small and large ribosomal subunits in *Δgno1* cells, the relative quantities of free 40S and 60S ribosomal subunits, 80S ribosomes and polysomes in total extracts from wild-type and *Δgno1* cells were determined after their differential sedimentation on a sucrose gradient (Figure 3A). The relative dimensions of the 40S and 60S peaks clearly indicate a deficit in free 60S ribosomal subunits relative to free 40S ribosomal subunits in *Δgno1* cells (Figure 3A, right panel). In addition, polysome abundance was vastly decreased in *Δgno1* cells, with the appearance of ‘shoulders’ on the 80S and the first and second polysome peaks, revealing the presence of so-called ‘half-mers’, i.e. mRNA-bound 40S subunits not associated with a 60S subunit. Such aberrant polysome profile is usually indicative of a 60S ribosomal subunit biogenesis defect. Importantly, expression of Gno1p-HA in *Δgno1* cells restored a normal ribosomal subunit and polysome accumulation profile (Figure 3B, bottom left panel). Expression of PINX1-HA in *Δgno1* cells also restored normal levels of 80S and polysomes but failed to fully restore levels of free 60S ribosomal subunits to normal (Figure 3B, bottom right panel).

**Figure 4.**
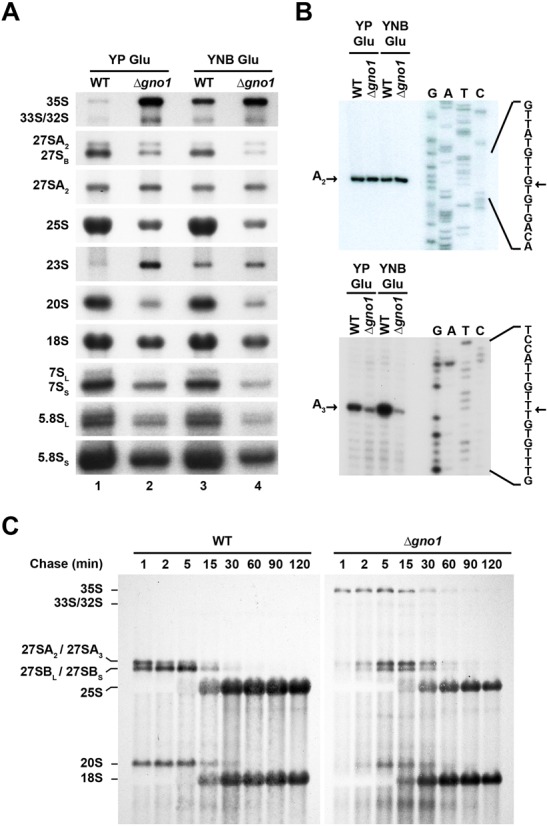
Analysis of pre-rRNA processing in *Δgno1* cells. (**A**) Northern blot analysis of various (pre)-rRNAs extracted from WT or *Δgno1* cells. RNAs were extracted from WT (lanes 1 and 3) or *Δgno1* (lanes 2 and 4) cells grown in YPGlu (lanes 1 and 2) or YNBGlu (lanes 3 and 4) media, separated on a 1.2% agarose gel in denaturing conditions, transferred to a nylon membrane and detected using various specific oligonucleotide probes. (**B**) Primer extension analysis of 27SA3 pre-rRNAs. Primer extensions were carried out using RNAs extracted from WT or *Δgno1* cells grown in YPGlu or YNBGlu media as indicated and a kinased primer hybridizing at the 3′ end of internal transcribed spacer 1 (5′TTAATATTTTAAAATTTCCAGTTACGAAAATTC3′). The sequencing ladder was generated using the same kinased primer. (**C**) Pulse-chase analysis. WT and *Δgno1* cells grown at 30°C in YNBGlu medium were incubated for 2 min with ^3^H-adenine. An excess of cold adenine was then added to the cultures and culture samples were collected at the indicated times after cold adenine addition. Total RNAs were extracted from these samples, separated on a 1.2% agarose gel in denaturing conditions and transferred to a nylon membrane. Labeled RNAs were directly detected by autoradiography.

Guglielmi and Werner have previously reported (29) increased steady-state levels of 35S pre-rRNA and impaired cleavages at the A_0_, A_1_ and A_2_ sites in *Δgno1* cells, resulting in lower 20S pre-rRNA accumulation (see Supplementary Figure S2 for a cartoon of pre-rRNA processing in yeast cells). They did not mention however a defect in the pre-60S pathway, except for a decrease in 27SA_2_ pre-rRNA levels as assessed by primer extension. We therefore carried out our own northern blot assessment of the relative accumulation levels of various pre-rRNA intermediates in *Δgno1* compared to wild-type cells (Figure 4A). As observed by Guglielmi and Werner, we detect an accumulation of the 35S pre-rRNA and a decrease in the steady-state levels of 20S pre-rRNA. There is also a very slight accumulation of the 33/32S pre-rRNAs. 23S pre-rRNA is increased when the *Δgno1* strain is grown in rich medium but not when it is grown in selective minimal medium. 27SA_2_ pre-rRNA levels are not diminished. The same conclusion can in fact be drawn from the northern analysis of Guglielmi and Werner. In contrast, levels of 27SA_3_ pre-rRNAs are decreased 2-fold in rich medium and 6-fold in minimal medium in *Δgno1* compared to wild-type cells as assessed by primer extension analyses (Figure 4B). In addition, steady-state levels of the 27SB pre-rRNAs are decreased 3-fold in rich medium and 4.6-fold in minimal medium in *Δgno1* compared to wild-type cells (Figure 4A). Moreover, levels of 7S_L_ and 7S_S_ pre-rRNAs are diminished 2.4-fold in rich medium and 3.5-fold in minimal medium, arguing that lack of Gno1p impairs the production and/or stability of both the long and short forms of 27SB. Primer extension analyses that detect pre-rRNA species, the 5′ end of which extends to sites B_1S_, B_1L_ and A_2_ (Supplementary Figure S3), are fully consistent with the northern data (Figure 4A). We conclude that Gno1p is required for the normal accumulation, and likely the normal production, of both pre-40S particles and intermediate nuclear pre-60S particles containing the 27SA_3_ or the 27SB_L_ pre-rRNA.

To explore the kinetics of pre-rRNA processing in *Δgno1* cells compared with wild-type cells, we performed pulse-chase analyses using ^3^H-adenine as a label (Figure 4C). These experiments demonstrate that processing of the 35S pre-rRNA is strongly delayed in *Δgno1* cells, this pre-rRNA being still detectable after 2 h of chase. Production of the 32S pre-rRNA is not inhibited but somewhat delayed. Production of the 27SA_2_ pre-rRNA is retarded, probably reflecting delayed 35S pre-rRNA processing. Production of 27SB pre-rRNAs is not completely inhibited but the pattern of accumulation and decrease of these pre-rRNAs is clearly aberrant. In wild-type cells, decrease in the levels of 27SA_2_ pre-rRNA is correlated with a transient increase in the levels of 27SB pre-rRNAs, such that after 2, 5 or even 15 min of chase, levels of labeled 27SB pre-rRNAs are higher than those of labeled 27SA_2_ pre-rRNAs. In *Δgno1* cells, in contrast, the decrease in the levels of labeled 27SA_2_ pre-rRNAs is accompanied by a decrease in the levels of 27SB pre-rRNAs, not by a transient increase, as in the wild-type situation. At no time point are the levels of labeled 27SB pre-rRNAs higher than those of labeled 27SA_2_ pre-rRNAs. Although the interpretation of these data is not straightforward, they may result from a combination of partial inhibition of 27SA_2_ pre-rRNA processing and partial turnover of the 27SB pre-rRNAs that are still produced. Production of 20S pre-rRNA is strongly inhibited in *Δgno1* cells, but the little 20S pre-rRNA that is produced can be converted to 18S rRNA. It is also quite clear that in *Δgno1* cells, the final accumulation of 18S rRNA is greater than that of 25S rRNA. Altogether, the results of the pulse-chase experiments are fully consistent with those of the polysome profile and northern analyses. The pulse-chase experiments confirm a major delay in 35S pre-rRNA processing, an imbalance in the accumulation of 25S rRNAs relative to 18S rRNAs and point to a defect in the production of 27SB pre-rRNAs that could be coupled with their increased turnover.

### Gno1p associates with 90S and early pre-60S particles in yeast

**Figure 5.**
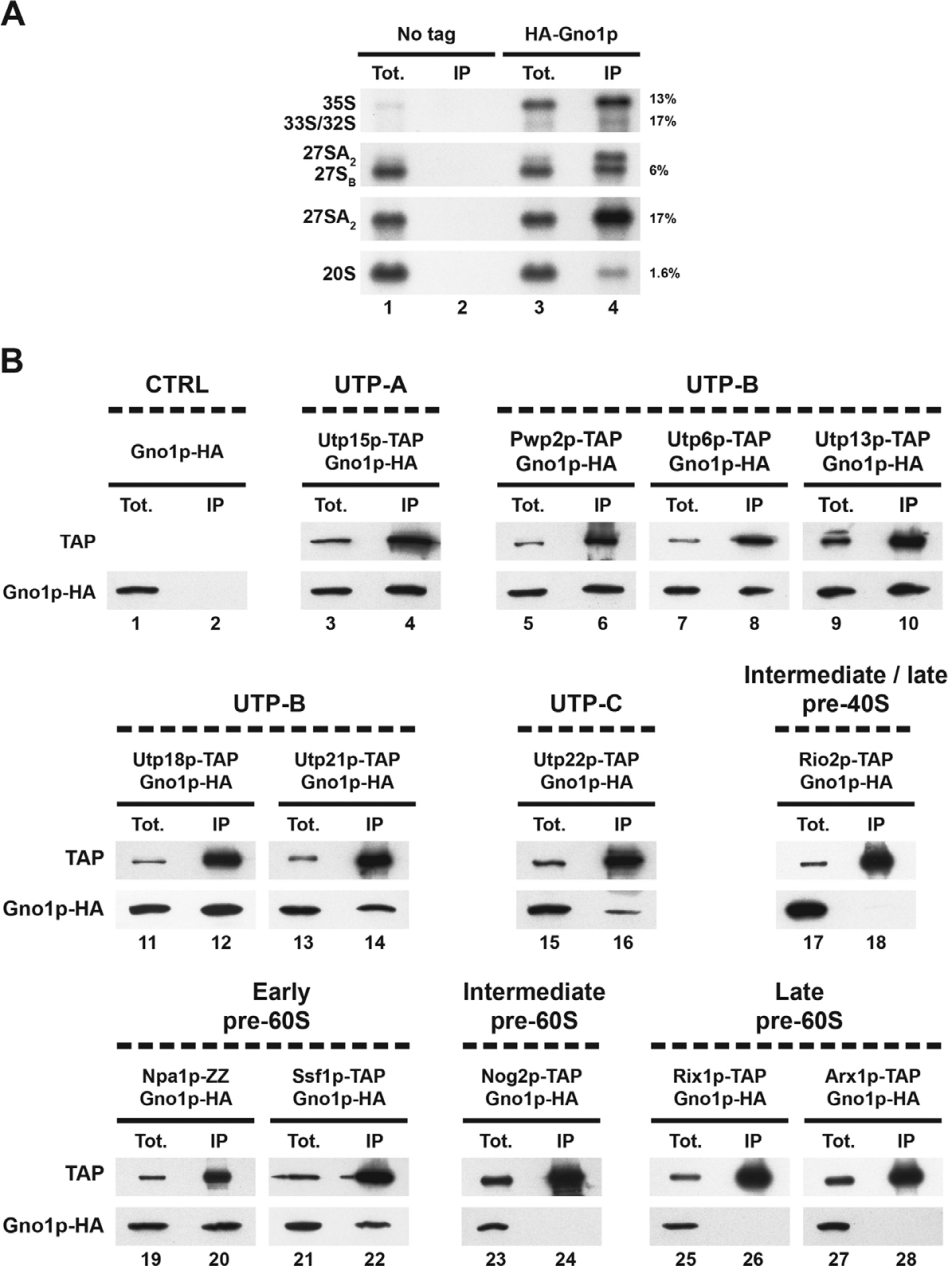
Immunoprecipitation experiments to assess the interactions of Gno1p-HA with specific components of different pre-ribosomal particles. (**A**) Interactions of Gno1p-HA with various pre-rRNAs. Gno1p-HA was precipitated from yeast extracts using anti-HA antibodies. Total RNAs were extracted from the pellets obtained following immunoprecipitation (lanes IP) or from 1/15th of the total input extracts used for precipitation (lanes Tot.), separated on a 1.2% agarose gel in denaturing conditions, transferred to a nylon membrane and detected using various specific oligonucleotide probes. The efficiency of precipitation (expressed as percentage of input) of a given pre-rRNA was determined using phosphorimager quantification and is indicated to the right of each panel. (**B**) Interactions of Gno1p-HA with protein components of various pre-ribosomal particles. Immunoprecipitation experiments have been carried out using IgG sepharose and extracts from cells expressing Gno1p-HA and, when indicated, a TAP- or ZZ-tagged component of a subset of pre-ribosomal particles. Proteins have been extracted from the pellets obtained following immunoprecipitation (lanes IP) or from 1/320th of the total input extracts used for precipitation (lanes Tot.) and analyzed by western as described in the legend of Figure 1A.

To help narrow down at which stage Gno1p acts during ribosome biogenesis in yeast, we precipitated HA-tagged Gno1p from total yeast extracts using anti-HA antibodies and identified the co-precipitated pre-rRNAs by northern analysis (Figure 5A). The most efficiently co-precipitated pre-rRNAs were the 33/32S and 27SA_2_ pre-rRNAs. 35S pre-rRNA was also significantly co-precipitated, while the 27SB and even more so the 20S pre-rRNAs were precipitated to a far lesser extent. We also assessed whether HA-tagged Gno1p can be co-precipitated with marker proteins of specific pre-ribosomal particles. Immunoprecipitation experiments were performed using IgG-sepharose and extracts from strains expressing HA-tagged Gno1p and a TAP-tagged component of a given subset of pre-ribosomal particles. Western analysis of the precipitated material using anti-HA antibodies shows that Gno1p-HA is co-precipitated together with components of the UTPA, UTPB and UTPC subcomplexes that sequentially assemble on the nascent pre-rRNA to nucleate assembly of the 90S particles (Figure 5B, lanes 3 to 16). Using the same approach, we could show that Gno1p-HA associates with Npa1p-ZZ and Ssf1p-TAP (Figure 5B, lanes 19 and 22), components of very early pre-60S particles containing 27SA_2_ pre-rRNA. In contrast, Gno1p-HA is not co-precipitated with Nog2p-TAP (Figure 5B, lanes 23 and 24), a protein that assembles within intermediate pre-60S particles containing 27SB pre-rRNA, nor is it associated with Rix1p-TAP (Figure 5B, lanes 25 and 26) or Arx1p-TAP (Figure 5B, lanes 27 and 28), components of late nucleoplasmic or cytoplasmic pre-60S particles. Gno1p-HA also failed to be co-precipitated with Rio2p-TAP, a component of late pre-40S particles (Figure 5B, lanes 17 and 18). Altogether, our data suggest that Gno1p is recruited within 90S pre-ribosomal particles and remains associated with early 27SA_2_ pre-rRNA-containing pre-60S pre-ribosomal particles. Our data further suggest that Gno1p displays only a transient association with pre-40S particles and that it dissociates from 27SB-containing intermediate pre-60S particles before the recruitment of Nog2p.

### The integrity of the G-patch domain of Gno1p or PINX1 is essential to their function during ribosome biogenesis in yeast

**Figure 6.**
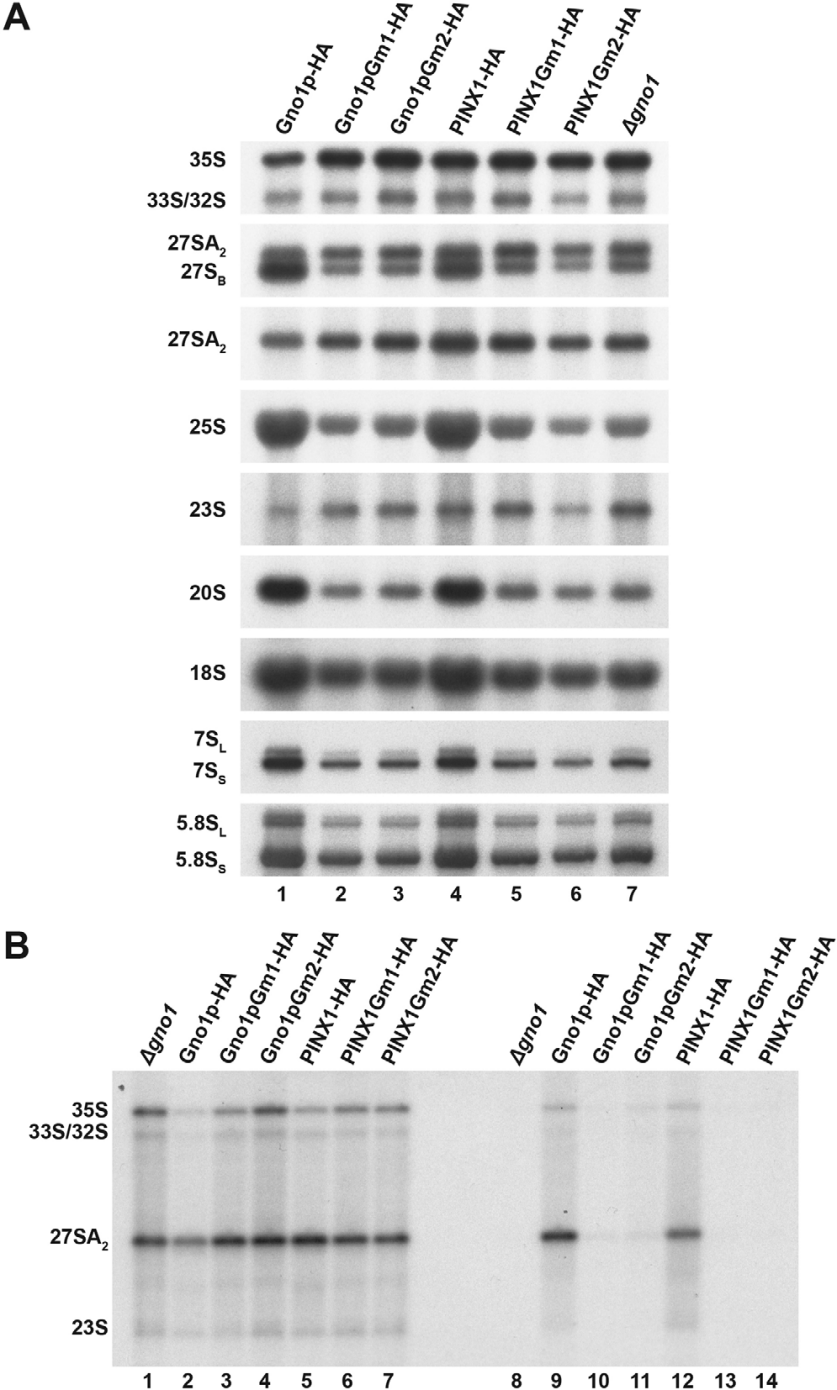
Consequences of alterations to the G-patch domain of Gno1p or PINX1 on ribosome biogenesis in yeast. (**A**) Northern analysis of rRNAs and pre-rRNAs extracted from *Δgno1* cells expressing Gno1p or PINX1 variants featuring amino acid substitutions within their G-patch domain. Total RNAs were extracted from *Δgno1* cells transformed with yeast vectors directing expression of wild-type Gno1p-HA (lane 1), Gno1pGm1-HA (lane 2), Gno1pGm2-HA (lane 3), PINX1-HA (lane 4), PINX1Gm1-HA (lane 5), PINX1Gm2-HA (lane 6) or the empty parental vector (lane 7) and analyzed by northern as described in the legend of Figure 4A. (**B**) Northern analysis of pre-rRNAs co-precipitated with HA-tagged Gno1p, PINX1 or G-patch variants thereof. Immunoprecipitation experiments were performed with an anti-HA matrix using extracts from *Δgno1* cells expressing the indicated HA-tagged protein or no tagged protein as control. Input (lanes 1 to 7) and co-precipitated (lanes 8 to 14) pre-rRNAs were analyzed as described in the legend of Figure 5A.

If Gno1p exerts its function during ribosome biogenesis mainly, or solely, via its interaction with Prp43p, we expect that disrupting this interaction would have the same effects on ribosome biogenesis as the total absence of the Gno1p protein. Although we still lack direct evidence for this assertion, we assume, based on the results obtained with PINX1, that Gno1p also directly interacts with Prp43p via its G-patch domain. We therefore assessed the effects on ribosome biogenesis of the amino acid substitutions within the G-patch domain of Gno1p or PINX1 which inhibit the Gno1p/Prp43p interactions in yeast and the PINX1/Prp43p interactions both in yeast and *in vitro* (see Figures 1B, C and 2B). To this end, we transformed the *Δgno1* strain with plasmids directing expression of Gno1pGm1-HA, Gno1pGm2-HA, PINX1Gm1-HA or PINX1Gm2-HA. As controls, the *Δgno1* strain was also transformed with the empty parental expression vector or vectors directing expression of wild-type HA-tagged Gno1p or PINX1. Western analysis demonstrated that Gno1pGm1-HA and Gno1pGm2-HA accumulate to the same levels as wild-type Gno1p-HA expressed from the plasmid, while the accumulation of PINX1Gm1-HA or PINX1Gm2-HA is somewhat diminished compared to that of PINX1-HA (Supplementary Figure S4A). Northern analysis of the pre-rRNAs and mature rRNAs extracted from these transformed strains demonstrated that expression of Gno1pGm1-HA, Gno1pGm2-HA, PINX1Gm1-HA or PINX1Gm2-HA completely fails to correct the mature rRNA and pre-rRNA accumulation defects (in particular that of 27SB pre-rRNAs) observed in the *Δgno1* strain transformed with the empty vector (Figure 6A). Immunoprecipitation experiments of HA-tagged proteins followed by western blot assessment of the co-precipitation efficiency of Prp43p confirm that G-patch alterations strongly weaken the association between Prp43p and Gno1p or PINX1 (Supplementary Figure S4B). Note that for reasons that are not clear, the Gno1pGm2-HA protein is precipitated far better than Gno1p-HA (Supplementary Figure S4B, lanes 9 and 11). Northern analysis of the co-precipitated RNAs shows that the weakened association between Prp43p and the altered versions of Gno1p or PINX1 is coupled with a weakened interaction of the latter proteins with the 35S, 32/33S and 27SA_2_ pre-rRNAs (Figure 6B). Collectively, these data lead us to conclude that these G-patch alterations completely abrogate the functionality of Gno1p and PINX1 during ribosome biogenesis in yeast.

## DISCUSSION

Yeast Pr43p is a multifunctional helicase, required for late spliceosome disassembly (16–18,35) and for the synthesis of both the small and large ribosomal subunits (19–21). In human cells, hPRP43, also called DHX15, is likewise involved in ribosome biogenesis: it is found in nucleoli (36) and its depletion leads to the nucleolar accumulation of the ribosomal protein Rps2 (37) and alterations in the levels of several pre-rRNAs (38). These alterations are compatible with processing defects at site 1 within the 5′ETS and sites 2 and C within ITS1. In addition, hPRP43/DHX15 has been found in nuclear speckles and was detected in pre-spliceosomal complex A and spliceosomal complex B (39,40). Thus, hPRP43/DHX15 is a splicing factor that could intervene at an early step of the splicing process, unlike its yeast counterpart involved at more downstream steps. Prp43p displays an intrinsically feeble ATPase activity *in vitro* (26), suggesting that *in vivo* it is activated by specific RNA substrates and/or co-factors during both splicing and ribosome biogenesis. Indeed, two yeast Prp43p-binding proteins have been identified, Ntr1p and Pfa1p, respectively, involved in spliceosome disassembly and pre-40S pre-ribosomal particle maturation, that can strongly stimulate its ATPase and/or helicase activities *in vitro* (24,26). In human cells, hPRP43/DHX15 interacts with the G-patch proteins RBM5 (41) and GPATCH2 (36) that activate the ATPase and, in the case of RBM5, the helicase activity of hPRP43/DHX15 *in vitro*. RBM5 is involved in the regulation of alternative splicing and could activate hPRP43/DHX15 during spliceosome assembly, since it is found mainly in pre-spliceosomal A complex (41). The process(es) in which GPATCH2 is involved remain to be characterized, but could be both splicing and ribosome biogenesis since GPATCH2 is found in nuclear speckles and nucleoli (36). We now show that both in yeast and human cells, Prp43p/hPRP43 interact with yet other G-patch proteins, namely the orthologuous Gno1p (yeast) and PINX1 (human) proteins. We demonstrate that PINX1 directly binds to yeast Prp43p. Gno1p most probably also directly interacts with Prp43p, although this will need to be demonstrated experimentally. Moreover, PINX1, like Pfa1p, RBM5 and GPATCH2, stimulates the ATPase activity of the helicase. Thus both in yeast and human cells, Prp43p/hPRP43 is stimulated by G-patch proteins involved in either splicing or ribosome biogenesis (maybe both in the case of GPATCH2). The ability to be stimulated by a G-patch protein is probably a characteristic of DEAH helicases, since another member of this family, namely Prp2p, physically and functionally interacts with the G-patch protein Spp2p (42). All these G-patch proteins are dissimilar apart from their G-patch domain. It is therefore unsurprising that in all cases examined, the G-patch domain seems to play an essential role in the direct interaction with the helicase and hence its activation (24,26,41). Our previous data further show that the C-terminal domain of Prp43p containing the OB-fold, a motif present in all DEAH RNA helicases, is required for Prp43p binding to the C-terminal domain of Pfa1p containing the G patch (43). It was also recently reported that a residue of Ntr1p G patch could be cross-linked to the C-terminus of Prp43p, lending strong support to the notion that Ntr1p G patch directly interacts with Prp43p C-terminal domain (44). It remains to be determined whether Prp43p interacts with all G-patch proteins via the same region. If this were the case, Prp43p could probably not interact with two different G-patch co-factors simultaneously. This proposal is supported by the fact that we failed to detect a physical interaction between Gno1p-HA and Pfa1p-TAP (Figure 1A). In contrast, Prp43p most probably can simultaneously interact with both RNA and a given G-patch protein since addition of RNA and a G-patch partner (Pfa1p-HIS or GST-PINX1-HIS) leads to a greater stimulation of the ATPase activity of Prp43p than either factor alone (Figure 2C and (26). How G-patch domains stimulate the ATPase activity of Prp43p remains little understood. The G-patch-containing N-terminal domain of Ntr1p alters the conformation of Prp43p OB-fold and ratchet domains and increases the efficiency of cross-linking between an RNA substrate and residues of Prp43p ratchet, RecA1 and RecA2 domains (44). These data argue that binding of Ntr1p G patch renders Prp43p nucleic acid-binding cavity more accessible. However, the action of G-patch proteins cannot be limited to facilitating the interaction with RNA since they increase Prp43p ATPase activity in the absence of RNA. Understanding their mode of action will require determining their atomic structure alone and in complex with Prp43p in the absence or presence of different additional ligands (RNA, ATP).

Our data confirm a previous report that Gno1p is required for efficient 35S pre-rRNA processing and normal accumulation of 20S pre-rRNA, the immediate precursor to 18S rRNA (29). These defects are accompanied by a decrease in the levels of both small and large ribosomal subunits (29). Our data further show that the 60S accumulation defect is due to a severe under-accumulation of the 27SA_3_ and 27SB pre-rRNAs (Figure 4), the RNA components of intermediate nuclear pre-60S particles. Levels of both the short and long forms of 7S pre-rRNAs are reduced to the same extent, indicating that the levels of their precursors, the short and long forms of 27SB pre-rRNAs, are affected equally. Accumulation of the precursor of 27SA_3_ and 27SB_L_ pre-rRNAs, namely 27SA_2_ pre-rRNA, is little affected by the absence of Gno1p (Figure 4 and Supplementary Figure S3). Our data therefore suggest that Gno1p is directly or indirectly required for 27SA_2_ pre-rRNA processing to 27SA_3_ and 27SB_L_ pre-rRNAs. Alternatively, or in addition, Gno1p could be required for the stability of 27SA_3_ and 27SB pre-rRNA-containing pre-60S particles. Gno1p associates mostly with 35S, 33/32S and 27SA_2_ pre-rRNAs, components of 90S and early pre-60S pre-ribosomal particles. Consistent with this finding, we detect an interaction between Gno1p and protein components of 90S particles and early pre-60S particles, but not with components of intermediate or late pre-60S particles, nor with Rio2p-TAP, a component of late pre-40S pre-ribosomal particles (Figure 5). The most straightforward interpretation of our data is that Gno1p is recruited to 90S pre-ribosomal particles, remains associated with early pre-60S particles and dissociates before recruitment of Nog2p within 27SB-containing intermediate nuclear pre-60S particles. This pattern of association with pre-ribosomal particles is fully consistent with a role of Gno1p in pre-40S particle as well as intermediate pre-60S pre-ribosomal particle formation and/or stability.

RNA interference-mediated depletion of PINX1 demonstrates that PINX1 is also involved in ribosome biogenesis in human cells (38). PINX1 depletion reduces 45S pre-rRNA accumulation, consistent with a defect in the assembly of early human pre-ribosomal particles. It also leads to a severe drop in the levels of 26S pre-rRNA, a precursor to 18S rRNA. The pattern of pre-rRNA accumulation defects seen in PINX1-depleted human cells is more subtle than the one obtained in yeast cells lacking Gno1p altogether. This difference may stem from residual levels of PINX1 after siRNA treatment.

The crucial issue is of course whether Gno1p or PINX1 requirement for pre-40S and intermediate pre-60S particle accumulation in yeast is due to their potential role as activators of the Prp43p helicase. If this were the case, we would expect Gno1p or PINX1 alterations inhibiting their interactions with Prp43p to produce a phenotype similar to the one caused by the absence of Gno1p. Hence we decided to investigate the functional consequences of amino acid substitutions in the G patch of Gno1p or PINX1 that inhibit the Gno1p/Prp43p interactions in yeast and the PINX1/Prp43p interactions both in yeast and *in vitro*. *Δgno1* yeast cells expressing such altered versions of Gno1p or PINX1 exhibit exactly the same aberrant pre-rRNA accumulation profile, with a severe 20S and 27SB pre-rRNA accumulation defect, as cells lacking Gno1p altogether (Figure 6). These data suggest that the 20S and 27SB pre-rRNA deficit is caused by a failure of Gno1p to bind to and therefore to activate Prp43p. We envisage that Gno1p could activate Prp43p within 90S pre-ribosomal particles and/or early pre-60S particles allowing Prp43p to drive conformational rearrangement(s) within these particles, required for the subsequent production and/or stability of pre-40S and intermediate pre-60S pre-ribosomal particles. Several Prp43p binding sites have been identified by the “UV Crosslinking and Analysis of cDNA” (CRAC) method within the 25S rRNA sequence (22), some of which have been linked to its role in releasing a subset of box C/D snoRNPs from the pre-rRNA. The remaining sites on the 25S rRNA sequence may well correspond to sites of interaction/action of the Prp43p/Gno1p holoenzyme required for 27SA_3_ and 27SB pre-rRNA formation or stability. It is also possible that the Prp43p/Gno1p complex is involved in snoRNP release in addition to its role in 27SA_3_/27SB pre-rRNA accumulation, although we have so far failed to find evidence for box C/D snoRNA retention within pre-ribosomal particles in the *Δgno1* strain.

Strikingly, the G-patch partner of hPRP43/DHX15 PINX1 was first characterized as a telomerase inhibitor (30). Yeast Gno1p was also proposed to act as a telomerase inhibitor (45), although this claim remains disputed (29). PINX1 is recruited to human telomeres via an interaction with the telomere-binding protein TRF1 (46) and inhibits telomerase by directly binding to the telomerase reverse transcriptase hTERT (30). The G-patch domain located close to the N-terminus of PINX1 (amino acids 24–69) seems dispensable for both PINX1-TRF1 and PINX1-hTERT interactions. Thus, in principle, PINX1 could be recruited to telomeres and interact with hTERT while bound to hPRP43/DHX15 via its G patch, leaving open the possibility of the involvement of this helicase in yet another fundamental cellular process, namely telomere length regulation.

## SUPPLEMENTARY DATA

Supplementary Data are available at NAR Online.

SUPPLEMENTARY DATA
