# Supplementary material for: The telomerase inhibitor Gno1p/PINX1 activates the helicase Prp43p during ribosome biogenesis
Source: Nucleic Acids Res. 2014 May 13;42(11):7330–45. doi: 10.1093/nar/gku357 (PMC4066782; doi:10.1093/nar/gku357)
Supplement: SUPPLEMENTARY DATA [file supp_42_11_7330__index.html]

The telomerase inhibitor Gno1p/PINX1 activates the helicase Prp43p during ribosome biogenesis — SUPPLEMENTARY DATA 

# The telomerase inhibitor Gno1p/PINX1 activates the helicase Prp43p during ribosome biogenesis

## SUPPLEMENTARY DATA

**Files in this Data Supplement:**

- SUPPLEMENTARY DATA
