# Supplementary material for: The telomerase inhibitor Gno1p/PINX1 activates the helicase Prp43p during ribosome biogenesis
Source: Nucleic Acids Res. 2014 May 13;42(11):7330–45. doi: 10.1093/nar/gku357 (PMC4066782; doi:10.1093/nar/gku357)
Supplement: SUPPLEMENTARY DATA [file supp_gku357_nar-03478-f-2013-File008.pdf]

SUPPLEMENTARY FIGURE LEGENDS

Supplementary Figure 1. Purified GST-ovoAC protein does not interact with purified Prp43p-HIS. Purified GST-ovoAC and Prp43p-HIS proteins were mixed and Prp43p-HIS was precipitated using anti-Prp43p antibodies as described in the Materials and Methods section. Proteins extracted from the pellet (lane IP) or TCA precipitated from the supernatant (lane Sup.) were subjected to SDS-PAGE and transferred to a nitrocellulose membrane. Prp43p-HIS was detected using anti-His antibodies and GST-ovoAC using anti-GST antibodies.

Supplementary Figure 2. Cartoon of major pre-rRNA processing steps and pre-rRNA intermediates in *Saccharomyces cerevisiae*.

Supplementary Figure 3. Primer extension analyses of pre-rRNAs extending to sites A_2, B_{1L} or B_{1S}. Primer extensions were carried out using RNAs extracted from WT or *Δgno1* cells grown in YNBGlu medium and a kinased primer hybridizing at the 5’ end of internal transcribed spacer 2 (5’GAGTATCACTCACTACGAAACAGAATGTTTG3’).

Supplementary Figure 4. (**A**) Western analysis of accumulation levels of Gno1p or PINX1 variants featuring amino acid substitutions within their G patch domain. Total proteins were extracted from *Δgno1* cells transformed with yeast vectors directing expression of wild-type Gno1p-HA (lane 1), Gno1pGm1-HA (lane 2), Gno1pGm2-HA (lane 3), PINX1-HA (lane 4), PINX1Gm1-HA (lane 5) or PINX1Gm2-HA (lane 6) and analysed by Western as described in the legend of Figure 1. Nhp2p used as loading control was detected using anti-Nhp2p antibodies. (**B**) Western analysis of the efficiency of Prp43p co-precipitation with HA-tagged Gno1p, PINX1 or G patch variants thereof. Immunoprecipitation experiments were performed with an anti-HA matrix using extracts from *Δgno1* cells expressing the indicated HA-tagged protein or no tagged protein as control. Proteins from input (lanes 1 to 7) and precipitated (IP, lanes 8 to 14) samples were analysed by Western as described in the legend of Figure 1A. HA-tagged proteins were detected using anti-HA antibodies and Prp43p using anti-Prp43p antibodies.

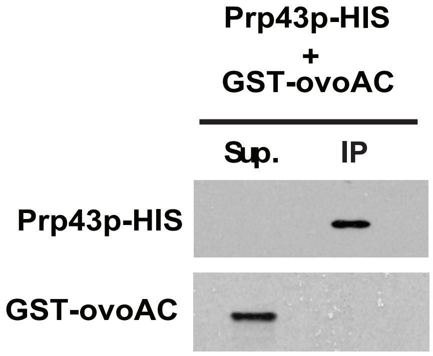

Supplementary Figure 1

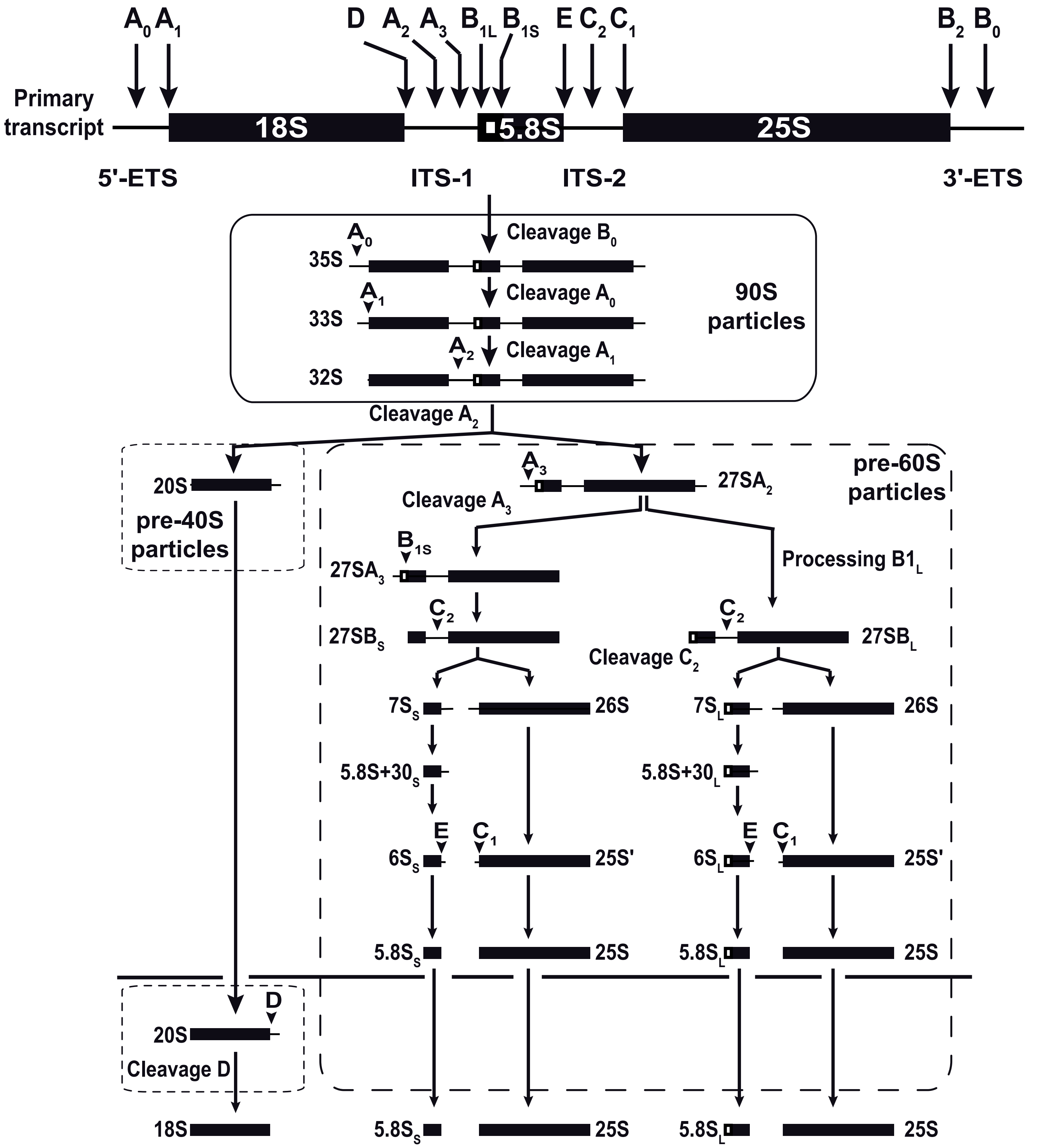

Supplementary Figure 2

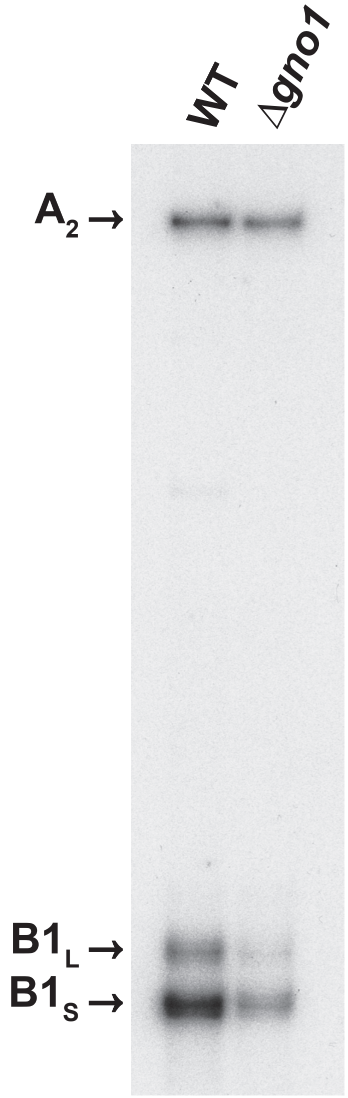

Supplementary Figure 3

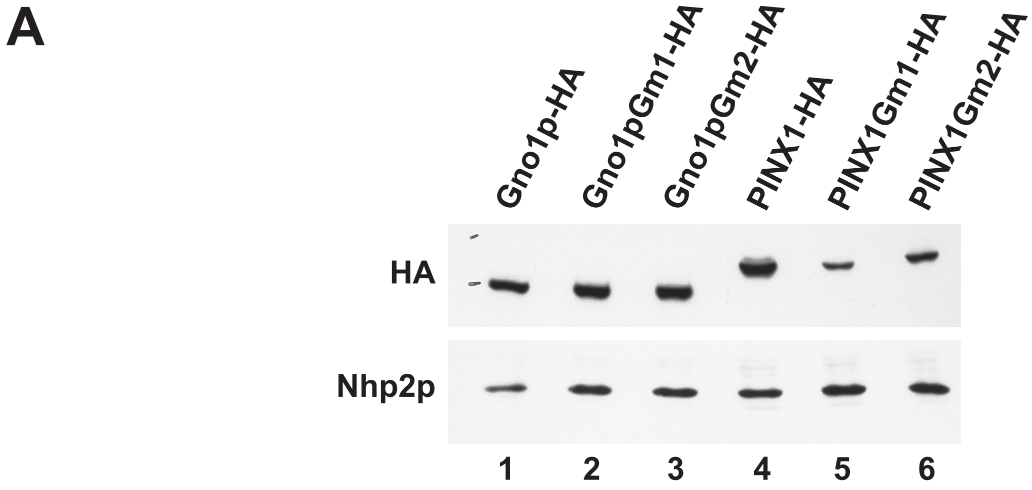

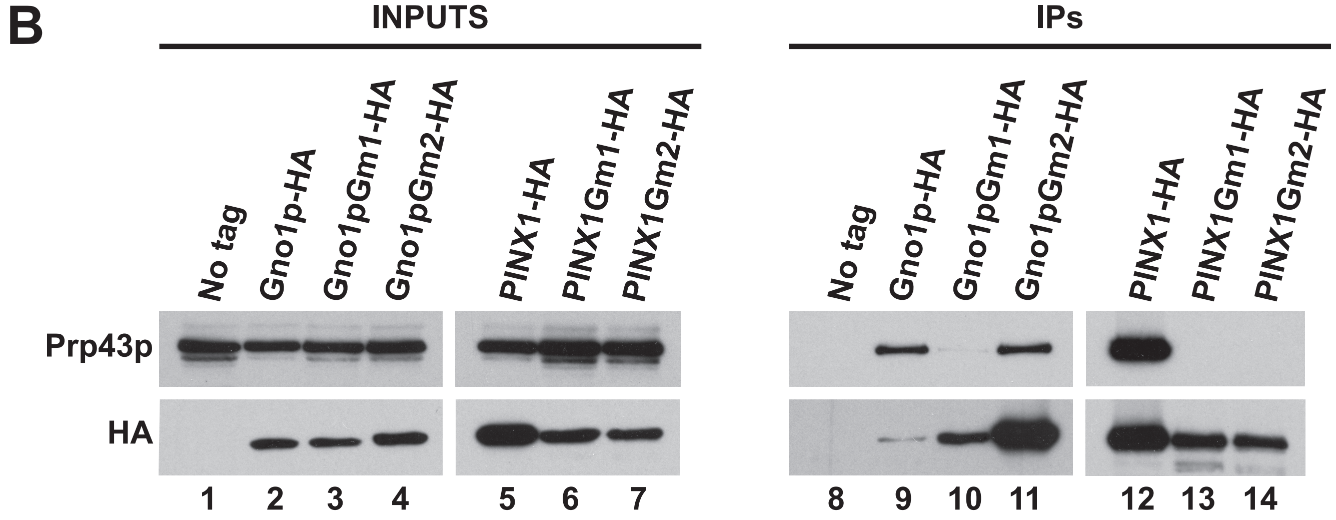

Supplementary Figure 4
